# Removal of Broken Cannulated Drill Bit

**DOI:** 10.7759/cureus.19706

**Published:** 2021-11-18

**Authors:** Khaled F Al-Kharouf, Kashif Abbas, Syed Anjum, Faisal I Khan

**Affiliations:** 1 Emergency Medicine, University Hospital Southampton NHS Foundation Trust, Southampton, GBR; 2 Trauma and Orthopaedics, University Hospital Southampton NHS Foundation Trust, Southampton, GBR

**Keywords:** orthopaedics, simple, safe, removal, remove, femoral, femur, bit, drill, broken

## Abstract

Breaking of surgical drill bits and subsequent dislodging in the bone are quite common in the field of orthopedics. Even though a few methods have been reported to remove dislodged drill bits, we present a novel method to remove a broken drill bit without additional instruments or a secondary incision.

A broken cannulated drill bit within the locking screw hole inside the neck of a femur was retrieved using a depth gauge with a curved tip that hooked onto the edge of the drill bit. By employing a clockwise and counter-clockwise twisting, the broken drill bit was retrieved through the proximal reaming tract. The 4mm tract, which was established by proximal reaming, immensely facilitated safe and time-efficient removal of the drill tip without further trauma or prolonging the surgery time. With our technique, the removal was simple and safe without further soft tissue trauma and blood loss. We advocate this approach for implementation in similar cases.

## Introduction

A surgical drill bit is used to create a cylindrical tunnel to accommodate a screw for rigid fixation. This tunnel prevents axial and shear forces from acting upon the screw and hence supports the load-bearing function of the skeleton during movement. Breaking an instrument during a surgical procedure is a challenging problem for any surgeon. In the world of orthopedics, a drill bit failure is not quite uncommon. Furthermore, the removal of the broken drill bit is often abandoned due to its difficult removal and prolonged surgical time. We describe a simple and useful technique that requires no additional instruments to facilitate the removal of a cannulated drill bit, and without prolonging surgical time or requiring secondary incisions.

## Technical report

**Figure 1:**
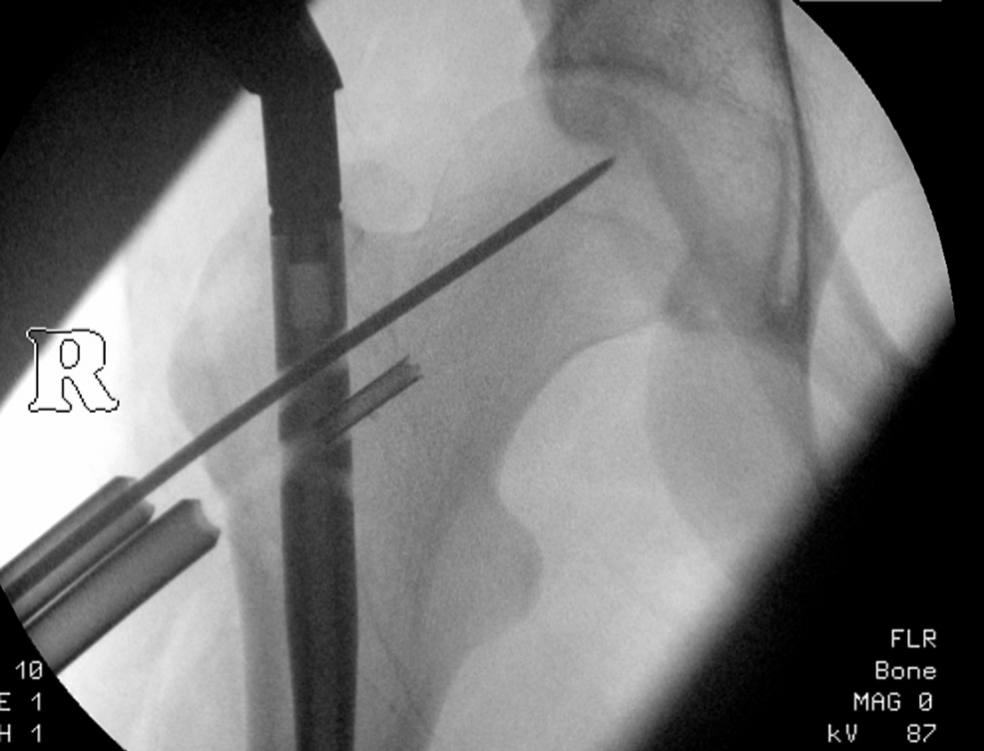
Broken drill bit

An 18-year-old young adult presented with a bilateral femoral shaft fracture following a road traffic accident. After reaming, the ante-grade femoral intramedullary nail was inserted. However, at the time of drilling for proximal fixation, a 4mm cannulated drill bit broke off and dislodged into the track (Figure 1). This is quite a common complication while drilling.

**Figure 2:**
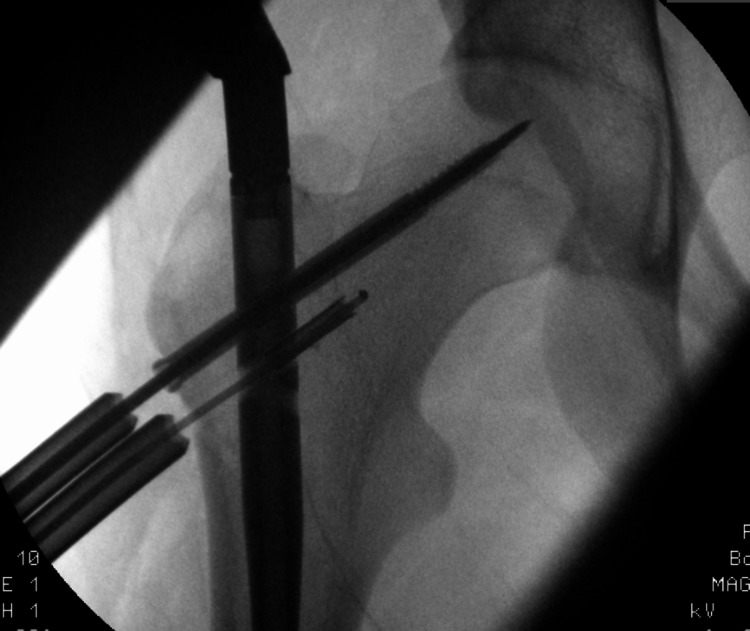
Inserting a depth gauge with a curved tip

In order to retrieve the broken drill bit, we used a depth gauge with a curved tip that hooked onto the edge of the drill bit (Figure 2).

**Figure 3:**
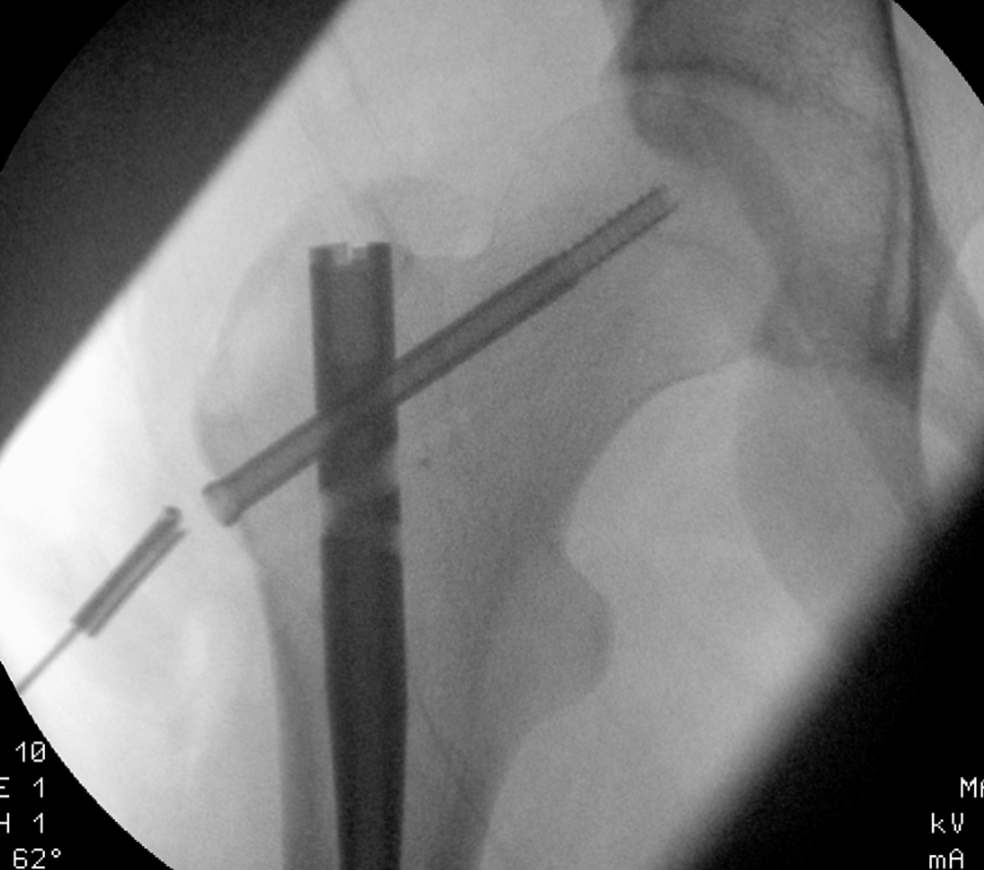
Retrieving the broken drill bit

Then, employing clockwise and counter-clockwise twisting, the broken drill bit was retrieved through the proximal reaming tract (Figure 3). The 4mm tract, which was established by proximal reaming, immensely facilitated safe and time-efficient removal of the drill tip without further trauma or prolonging the surgery time.

## Discussion

In the world of orthopedics, breakage or failure of instruments is quite common. Most frequently, drill bits, Kirschner wires, and guide pins are known to break or fracture during insertion [1]. These bits and pieces if left inside the body can migrate, cause infection, damage the cartilage or, may interfere with planned hardware placement. Even though in some instances surgeons have left broken hardware inside the body, it is a risk to the surrounding blood vessels and nerves and poses the threat of migration. In one instance, a drill bit was left inside after a total disc replacement and ended up traveling into the spinal canal by the one-year postoperative follow-up [2]. As illustrated by Pichler et al. [1], most cases of instrument breakage in orthopedic procedures do not cause serious problems, which is supported by data from 11,856 procedures undertaken in two hospitals. As per the data, the broken instrument was left inside the body in 37 patients, and none of these patients showed any symptoms during the follow-up period. Even though postoperative surgical complications arising from broken instruments are rare, a broken drill bit ending up in the spinal cord after the one-year postoperative follow-up required further surgery to have it removed [2]. In an effort to reduce postoperative complications, we strongly encourage the safe and simple removal of broken drill bits during the initial operation.

Chouhan and Sharma [3] describe a push-back technique for cases where the length of the broken drill bit is greater than the diameter of the bone for both proximal femur and distal femur. Other methods of retrieving broken drill bits include either using K-wires [4] or new screws [5] to push the drill bit to the opposite side, requiring a new incision on the other side. These methods are more challenging because a lot of soft tissue dissection is involved. In contrast, the Chouhan and Sharma [3] push-back technique is a much safer alternative to using K-wires or new screws. However, it involves pushing the drill bit by a narrow blade Langenbeck retractor, which may fail in cases where a shorter drill bit is confined inside the medullary canal of the femur. The push-back technique requires the drill bit to be longer in length than the diameter of the femur. Our method enables us to remove a shorter cannulated drill bit via a depth gauge even in instances where the drill bit is retained inside the medullary canal and is shorter in length than the diameter of the femur. 

Bassi et al. [6] has promoted the Bassi technique to remove larger cannulated drill bits of 4mm diameter or more, which also involves using a smaller number of K-Wires. In comparison, our technique consists of inserting a depth gauge with a curved hook into the proximal reaming tract and subsequently latching the hook onto the edge of the cannulated drill bit. Then, a clockwise and anti-clockwise twisting motion of the depth gauge retrieves the broken drill bit. One pitfall of our method is that it only applies to cannulated drill bits, which facilitates latching of the curved tip of the depth gauge to hook on to the drill bit. This would not be possible on solid drill bits. We believe using a depth gauge to retrieve broken cannulated drill bits is more time-efficient and less traumatic in contrast to the Bassi Technique [6], or using further K-wires [4], additional screws [5], and the push-back technique [3]. As surgeons, we need to be aware of all these methods while dealing with challenging situations.

## Conclusions

Our method does not require any additional instrument and is much simpler to perform as long as a proximal reaming tract is intact to help facilitate the utilization of a depth gauge with a curved tip to retrieve the broken cannulated drill bit through the original incision. With our technique, the removal was simple and safe without further soft tissue trauma and blood loss. We advocate this approach for similar cases.
